# The effect of succinate on brain NADH/NAD^+^ redox state and high energy phosphate metabolism in acute traumatic brain injury

**DOI:** 10.1038/s41598-018-29255-3

**Published:** 2018-07-24

**Authors:** Matthew G. Stovell, Marius O. Mada, Adel Helmy, T. Adrian Carpenter, Eric P. Thelin, Jiun-Lin Yan, Mathew R. Guilfoyle, Ibrahim Jalloh, Duncan J. Howe, Peter Grice, Andrew Mason, Susan Giorgi-Coll, Clare N. Gallagher, Michael P. Murphy, David K. Menon, Peter J. Hutchinson, Keri L. H. Carpenter

**Affiliations:** 1 Division of Neurosurgery, Department of Clinical Neurosciences, University of Cambridge, Cambridge, UK; 2 Wolfson Brain Imaging Centre, Department of Clinical Neurosciences, University of Cambridge, Cambridge, UK; 3 Department of Clinical Neuroscience, Karolinska Institutet, Stockholm, Sweden; 4 Department of Neurosurgery, Keelung Chang Gung Memorial Hospital, Chang Gung University College of Medicine, Taoyuan, Taiwan; 5 Department of Chemistry, University of Cambridge, Cambridge, UK; 6 Division of Neurosurgery, Department of Clinical Neurosciences, University of Calgary, Calgary, Canada; 7 MRC Mitochondrial Biology Unit, University of Cambridge, Cambridge, UK; 8 Division of Anaesthesia, Department of Medicine, University of Cambridge, Cambridge, UK

## Abstract

A key pathophysiological process and therapeutic target in the critical early post-injury period of traumatic brain injury (TBI) is cell mitochondrial dysfunction; characterised by elevation of brain lactate/pyruvate (L/P) ratio in the absence of hypoxia. We previously showed that succinate can improve brain extracellular chemistry in acute TBI, but it was not clear if this translates to a change in downstream energy metabolism. We studied the effect of microdialysis-delivered succinate on brain energy state (phosphocreatine/ATP ratio (PCr/ATP)) with ^31^P MRS at 3T, and tissue NADH/NAD^+^ redox state using microdialysis (L/P ratio) in eight patients with acute major TBI (mean 7 days). Succinate perfusion was associated with increased extracellular pyruvate (+26%, p < 0.0001) and decreased L/P ratio (−13%, p < 0.0001) in patients overall (baseline-vs-supplementation over time), but no clear-cut change in ^31^P MRS PCr/ATP existed in our cohort (p > 0.4, supplemented-voxel-vs-contralateral voxel). However, the percentage decrease in L/P ratio for each patient following succinate perfusion correlated significantly with their percentage increase in PCr/ATP ratio (Spearman's rank correlation, r = −0.86, p = 0.024). Our findings support the interpretation that L/P ratio is linked to brain energy state, and that succinate may support brain energy metabolism in select TBI patients suffering from mitochondrial dysfunction.

## Introduction

Following traumatic brain injury (TBI) a series of damaging pathophysiological processes occur in the brain that may lead to death or disability. A key component of these events is brain metabolic failure. This can encompass a range of pathological processes including classical ischaemia (lack of blood supply, which may stem from raised intracranial pressure or hypotension), hypoxia and increased tissue diffusion barrier. If this energy failure occurs in the setting of normal brain oxygenation and glucose supply it is commonly termed mitochondrial dysfunction^1,2^.

We have recently shown that succinate delivered by microdialysis to the injured brain lowers the lactate/pyruvate (L/P) ratio, which is understood to reflect an improvement in cellular NADH/NAD^+^ redox state^3,4^. We suggested that succinate therapy achieves this by supporting mitochondrial TCA cycle function, which in turn can improve brain energy metabolism. However, it has not yet been demonstrated empirically that improved L/P ratio translates to better cellular energetics and is associated with favourable changes in brain high energy phosphates – adenosine triphosphate (ATP) and its high-energy reserve species phosphocreatine (PCr).

*In-vivo* ^31^P Magnetic Resonance Spectroscopy (MRS) allows measurement of these high energy phosphates in the living brain. Changes in the ratio PCr/ATP, assessed using ^31^P MRS, have been noted in various cerebral pathologies^5–10^ but we are the first to report ^31^P MRS in TBI patients who require sedation, intubation and invasive intracranial monitoring for control of intracranial hypertension, and with measurements of extracellular biochemistry.

In this study, we measured the ratio of brain PCr/ATP acutely (within the first 10 days following injury) in patients suffering from traumatic brain injury using *in-vivo* ^31^P Magnetic Resonance Spectroscopy (^31^P MRS) after microdialysis delivery of succinate to establish if changes in L/P ratio are accompanied by changes in brain high energy phosphate metabolism. Brain intracellular pH was also evaluated using ^31^P MRS; as an indicator of core physiology.

## Results

### Patient demographics

**Figure 1:**
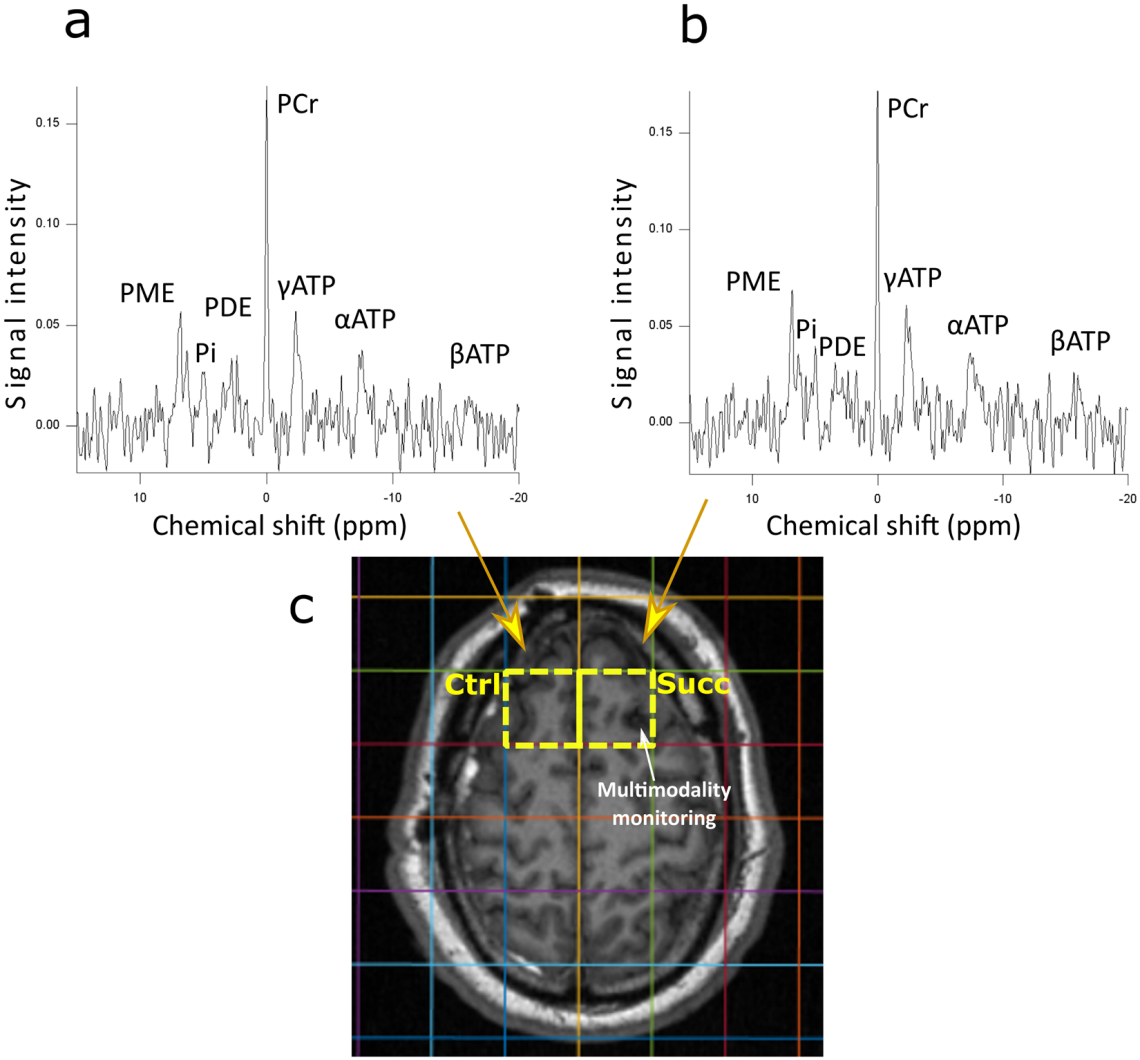
Example ^31^P MRS spectra (**a** and **b**) from a patient suffering from acute major TBI, acquired using a custom head-coil (Pulseteq Ltd) and 3T Siemens scanner. Axial magnetization-prepared rapid gradient-echo (MP-RAGE) MRI scan (**c**) demonstrates voxel origin within the CSI grid. The patient’s left frontal voxel (yellow border, marked ‘Succ’ for succinate, on the right of the image) contains a microdialysis catheter perfused with 12 mmol/litre disodium 2,3-^13^C_2_ succinate for 24 hours directly before the MR scan (spectrum shown in **b**). The patient’s right frontal voxel (yellow border, marked ‘Ctrl’ for control, on the left of the image) has no microdialysis catheter or supplementation and was used as the paired control (spectrum shown in **a**). Key metabolites are annotated. PME: phosphomonoesters, Pi: inorganic phosphate, PDE: phosphodiesters, PCr: phosphocreatine, ATP: adenosine triphosphate. For further details see Methods section.

Eight patients suffering from TBI received focal disodium succinate (12 mmol/L) perfusion for 24 hours via a frontal microdialysis catheter (Table 1) and a preceding/succeeding baseline period of microdialysis with normal CNS perfusion fluid. Of the 8 patients, 7 had *in-vivo* ^31^P MRS that yielded spectra with acceptable signal-to-noise ratio for analysis. Example scan and spectra are shown in Fig. 1.

**Table 1:** Patient demography.

Patient	Age (years)	Sex	Injury Mechanism	Brain Injury	Admission GCS	Days from TBI	Catheter insertion
A	62	M	Presumed assault	ASDH, brain contusions	10	7	R-CAD
B	63	F	Fall from height	ASDH, ICH	7	3	R-T
C	24	F	RTC	EDH, brain contusions	10	4	R-CAD
D	65	M	RTC	brain contusions	6	6	L-T
E	51	M	RTC	EDH, ICH	3	4	R-T
F	42	M	Assault	DAI, early hypoxia*	8	10	L-CAD
G	29	M	Assault	EDH, brain contusions	8	5	L-CAD
H	42	F	RTC	ASDH, brain contusions	3	3	L-T

M: male, F: female, RTC: road traffic collision, GCS: Glasgow Coma Scale, EDH: extradural hematoma, ASDH: acute subdural hematoma, ICH: intracerebral hematoma. GCS denotes highest GCS at presentation to emergency services. Catheter insertion denotes side (R/L) and whether via cranial access device (CAD) or tunnelled (T) at time of craniotomy/craniectomy. The catheters were not directed into, nor adjacent to, lesions identified on computerized tomography (CT). Patients A and C presented only moderately drowsy but then rapidly deteriorated; requiring sedation, intubation, ventilation and surgery for their TBI followed by a period of intracranial multimodality monitoring and treatment for intracranial hypertension. Patients A and F had persistently high ICP and were scanned as soon as they could tolerate lying flat. *Patient F had suspected hypoxia at the assault scene, but not while in neurocritical care unit. All patients received microdialysis perfusion with succinate and had ^31^P MRS; in one case (H) the ^31^P data were unusable due to low signal-to-noise.

### Succinate supplementation resulted in an improved (lowered) lactate/pyruvate ratio

**Figure 2:**
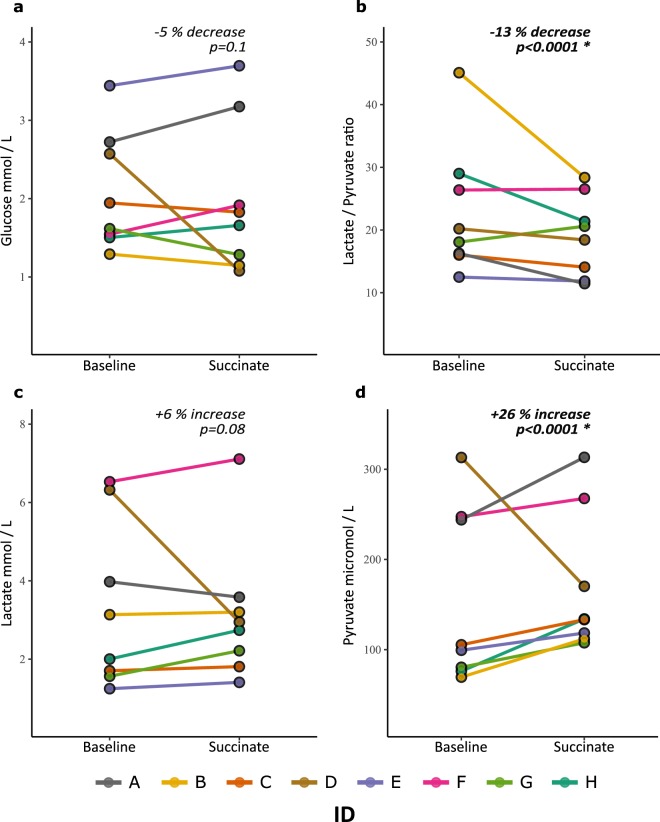
Line-plots of microdialysis measurements measured by bedside ISCUS: (**a**) glucose; (**b**) lactate/pyruvate ratio (L/P ratio); (**c**) lactate; (**d**) pyruvate. Individual subject means from a period of 12 mmol/L succinate perfusion (24 hours) and preceding/succeeding baseline perfusion (24 hours) are represented. There was a statistically significant 13% (mean of subject means) fall in lactate/pyruvate ratio (p < 0.0001) and a statistically significant 26% (mean of subject means) rise in pyruvate (p < 0.0001) analysed using lmer in R. For further details see Methods and Results sections.

Comparing the period of baseline perfusion with the period of succinate perfusion, we found results similar to those in a subset of our previous cohort (Jalloh *et al*.^3^) (Table 2) (Fig. 2). Following succinate supplementation, there was a statistically significant (by linear mixed effect model) mean 13% decrease in L/P ratio (p < 0.0001, decrease in 6/8 patients and increase in 2/8), caused by a mean 25% increase in pyruvate (p < 0.0001, increase in 7/8 patients and decrease in 1/8). There was no statistically significant change in lactate, with mean increase 6% (p = 0.08, increase in 6/8 patients and decrease in 2/8) or glucose, with mean decrease 5% (p = 0.1, decrease in 4/8 patients and increase in 2/8). In patients A, B and H the decrease in L/P ratio was considerable (>26%), furthermore two of these patients (B and H) had high baseline L/P ratios of 45.1 and 29 respectively. Brain tissue oxygen (PbtO_2_) data were available for 4 patients, whose levels were all 25 mmHg or above, thus none of these 4 individuals suffered from brain hypoxia during the study. Three patients did not have PbtO_2_ monitoring, and one patient’s PbtO_2_ data was not available. More detailed PbtO_2_, intracranial pressure (ICP) and cerebral perfusion pressure (CPP) data can be found in Supplementary Table S2.

**Table 2:** Results of microdialysis measured by ISCUS for Patients A-H.

Metabolite	Condition	A	B	C	D	E	F	G	H	Mean change
L/P ratio	Baseline	16.3	45.1	16.0	20.2	12.5	26.4	18.1	29.0	**−13**% ***p*** < ***0.0001***
	Succinate	11.5	28.4	14.1	18.4	11.8	26.5	20.6	21.4	
	% change	−29.6	−37.1	−12.1	−8.8	−5.2	+0.6	+14.0	−26.4	
Glucose (mM)	Baseline	2.7	1.3	1.9	2.6	3.4	1.5	1.6	1.5	−5% *p* = *0.1*
	Succinate	3.2	1.1	1.8	1.1	3.7	1.9	1.3	1.7	
	% change	+16.6	+11.3	−6.1	−58.1	+7.4	+23.9	−20.5	+10.3	
Lactate (mM)	Baseline	4.0	3.1	1.7	6.3	1.2	6.5	1.6	2.0	+6% *p* = *0.08*
	Succinate	3.6	3.2	1.8	2.9	1.4	7.1	2.2	2.7	
	% change	−9.9	+2.1	+6.2	−53.4	+13.0	+8.9	+41.6	+36.7	
Pyruvate (μM)	Baseline	244	70	105	313	99	247	81	76	**+26**% ***p*** < ***0.0001***
	Succinate	313	112	133	170	118	268	108	134	
	% change	+28.4	+60.9	+26.4	−45.6	+19.2	+8.1	+33.8	+75.9	

Mean results of microdialysis samples analysed with ISCUS during baseline perfusion with normal perfusion fluid or a period with succinate supplemented perfusion fluid. L/P ratio: lactate/pyruvate ratio, mM: millimole/litre, μM: micromole/litre. *p-values* from analysis of pooled results using the package lmer in R, (significance <0.05 denoted by bold font) - see Methods subsection Statistical analysis for further details.

### Decrease of Lactate/Pyruvate ratio was associated with an increased PCr/γATP ratio

**Figure 3:**
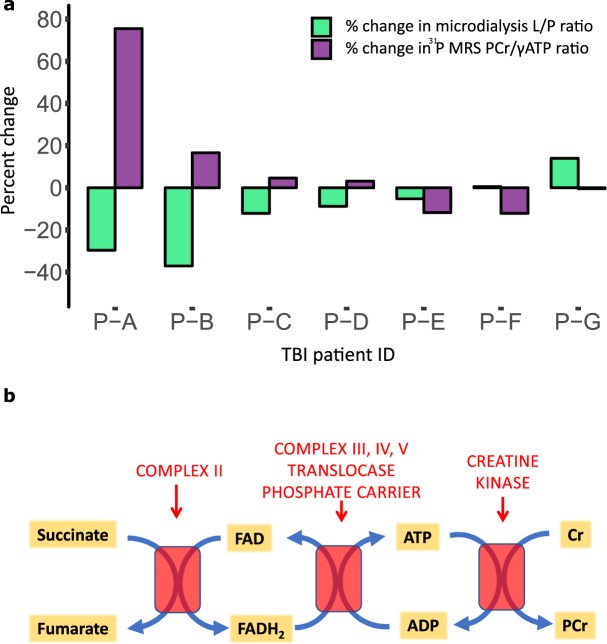
Bar chart (**a**) showing the inverse relationship between percentage change in brain microdialysate L/P ratio (for succinate perfusion compared with a baseline unsupplemented perfusion period in the same catheter), and percentage difference in PCr/γATP (for a voxel that had received succinate perfusion compared to a partner control contralateral voxel without succinate). This inverse relationship was statistically significant (Spearman’s r = −0.86, p = 0.024). Thus, a greater percentage reduction in the L/P ratio correlates with a greater percentage increase in the PCr/γATP ratio. (**b**) Simplified schematic showing potential mechanism of succinate’s effect on PCr/ATP. Succinate misses out Complex I of the mitochondrial electron transport chain. Succinate’s oxidation to fumarate by Complex II (succinate dehydrogenase) reduces FAD to FADH_2_ – which is oxidized back to FAD – releasing electrons that pass through a chain of oxidation/reduction reactions, complex II to CoQ, complex III, Cytochrome C and complex IV with molecular oxygen as terminal electron acceptor, culminating in conversion of oxygen to water, while complexes III and IV export protons across the mitochondrial inner membrane creating a proton electrochemical potential gradient, driving ATP synthesis at complex V (ATP synthase), converting ADP to ATP. In the mitochondrial intermembrane space ATP donates its high energy phosphate to creatine, producing ADP and PCr, which diffuses into the cell cytoplasm for use by cellular machinery.

There was an inverse relationship between the improvement (lowering) in L/P ratio following succinate supplementation with the increase in PCr/γATP, comparing the voxel receiving the succinate and its ‘control’ contralateral partner voxel that did not receive succinate, when these changes in PCr/ATP and L/P ratio were expressed as percentages (Fig. 3a; Spearman's rank correlation, r = −0.86, p = 0.024).

### ^31^P MRS revealed no difference in PCr/γATP ratio or intracellular pH in cohort overall

**Figure 4:**
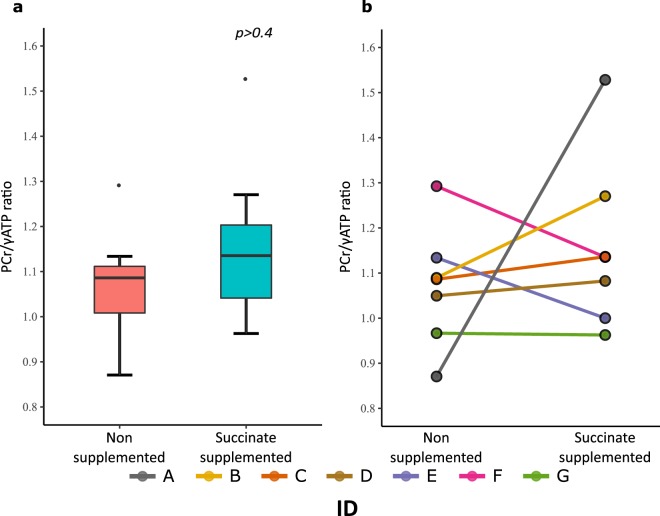
^31^P MRS PCr/γATP boxplot (**a**) and lineplot (**b**) of frontal voxels that received microdialysis delivery of 12 mmol/L succinate and their corresponding partner (contralateral) frontal voxels that did not receive succinate. There were no significant differences in PCr/γATP between succinate supplemented voxels and their contralateral voxel without succinate (p > 0.4, paired comparison using Wilcoxon signed rank test). The two voxels were analysed within the same MRI scan, in each patient.

The frontal voxels supplemented with succinate by their microdialysis catheters were compared with their adjacent contralateral equivalent frontal voxels that did not receive succinate supplementation (‘partner’ voxel). We could not detect a statistically significant overall difference in PCr/γATP ratio (Fig. 4) between the voxels that received focal succinate perfusion and their contralateral equivalent frontal ‘partner’ voxel (Wilcoxon signed rank test, p > 0.5). While the group level did not show an overall significant difference, some of these patients had a markedly higher PCr/γATP levels following succinate supplementation (Table 3). Also, we have demonstrated (see above) a statistically significant relationship between percentage increase in PCr/γATP and percentage decrease in extracellular L/P ratio (Fig. 3a).

**Table 3:** PCr/γATP ratio results from ^31^P MRS analysis of frontal voxels supplemented with succinate, compared simultaneously to their contralateral unsupplemented voxels.

Subject I.D.	PCr/γATP ratio Unsupplemented (contralateral) voxel	PCr/γATP ratio Succinate-supplemented voxel	Percentage difference
A	0.871	1.528	+75.5%
B	1.089	1.270	+16.6%
C	1.086	1.136	+4.6%
D	1.050	1.082	+3.1%
E	1.134	1.000	−11.9%
F	1.293	1.135	−12.2%
G	0.967	0.963	−0.4%

^31^P MRS measurements of PCr/γATP in the frontal voxels of the seven patients who received succinate supplementation. Each voxel that received succinate was matched with a partner contralateral frontal voxel that did not receive supplementation, within the same patient. The difference in PCr/γATP between supplemented and matched unsupplemented voxels was not statistically significantly different (two-tailed Wilcoxon signed rank test *p* = *0.58*). An eight patient (H) also underwent ^31^P MRS but yielded no useable data due to low signal-to-noise.

^31^P MRS revealed no statistically significant difference in brain intracellular pH in the cohort overall as a result of succinate supplementation via microdialysis vs. contralateral ‘partner’ voxel without succinate (Wilcoxon signed rank test, p = 1). Intracellular pH values are presented in Supplementary Table S1. There was no statistically significant relationship between percentage change in extracellular L/P ratio and either percentage change in brain intracellular pH or absolute change in brain intracellular pH.

## Discussion

We demonstrate the first study of human brain metabolism in TBI that combines microdialysis and *in-vivo* ^31^P MRS – comparing the effect of succinate supplementation on brain metabolism using both modalities.

We have shown that succinate improves NADH/NAD^+^ redox state (decreases L/P ratio) in the traumatised human brain. While we did not detect an overall statistically significant change in high energy phosphates (PCr/ATP) after succinate supplementation in the cohort, those individuals who had the greatest improvement (decrease) in L/P ratio after succinate perfusion – i.e. those suffering from mitochondrial dysfunction – had considerable increases in their PCr/ATP ratio measured using ^31^P MRS.

We suggest that the intrinsic heterogeneity of TBI may have been a factor leading to the absence of a clear-cut overall difference in PCr/ATP between a succinate-supplemented voxel and its paired contralateral unsupplemented voxel when measured simultaneously by ^31^P MRS in this small cohort of patients. Microdialysis measurements, contrastingly, were performed within-voxel over a course of time and showed significant differences across the cohort between an unsupplemented baseline perfusion period and a succinate-supplemented perfusion period – notably improvement (lowering) of L/P ratio. Moreover, our finding of a significant correlation within the cohort between percentage decrease in L/P ratio and percentage increase in PCr/ATP suggests that succinate supplementation can increase cerebral high-energy phosphate status. This extends previous evidence that succinate delivered by microdialysis is metabolised by the TCA cycle^3^.

The oxidation of succinate to fumarate is unique as it is both a key step of the TCA cycle and the first step of the electron transport chain for the conversion of flavin adenine dinucleotide (FAD) to its reduced form FADH_2_. It is catalysed by succinate dehydrogenase – which is Complex II of the electron transport chain. There is evidence that Complex II is less susceptible to injury than Complex I which oxidises nicotinamide adenine dinucleotide (NADH), so succinate has been proposed as a substrate that may support the TCA cycle and ATP generation by oxidative phosphorylation in stressed cells when mitochondrial function is impaired^3,11^.

Succinate is unlikely to benefit brain metabolism in cases of hypoxia or ischaemia. Without oxygen, the mitochondrial electron transport chain cannot function properly. In experimental ischaemia, succinate dehydrogenase (electron transport chain complex II and part of the TCA cycle) was reported to run ‘backwards’, building up succinate and reverse electron transport to complex I^12^. In that study, reperfusion produced a surge in reactive oxygen species (via complex I) and cell death. The present study did not involve ischaemia–reperfusion. We have previously suggested that using succinate to support mitochondrial metabolism should be performed in the presence of adequate oxygenation, not during ischaemia–reperfusion^3^. None of the four patients in the present study with available Licox PbtO_2_ data suffered from brain tissue hypoxia during the monitoring period, therefore we think it unlikely that this affected the other four patients in our study, as they received similar critical care management which specifically aims to avoid this. Furthermore, the ISCUS data suggests none of the 8 patients studied were ischaemic as microdialysate glucose levels were above 1 mmol/L in each case.

Previously we have shown that 2,3-^13^C_2_ succinate delivered via microdialysis enters the TCA cycle of the traumatised brain, demonstrated by the detection of downstream ^13^C labelled metabolites in the collected microdialysates using high-resolution ^13^C NMR^3^. Succinate uptake into cells can occur via the SLC13 family of Na^+^-coupled di-carboxylate and tri-carboxylate transporters^13–15^. SLC13 occur widely, including in brain, astrocytes and neurons where succinate uptake and metabolism were also shown using radiolabelling^16–18^. Additionally, nonspecific uptake might occur in any cells with increased plasma membrane permeability. Appearance of metabolites with characteristic ^13^C NMR doublet signals clearly indicated uptake and mitochondrial metabolism of 2,3-^13^C_2_ succinate^3^. The doubly ^13^C-labelled metabolites were unambiguous evidence the 2,3-^13^C_2_ succinate molecules crossed from the perfusate into the brain extracellular space, entered the cells and were metabolised, exported into the extracellular fluid and recovered by the microdialysis catheter^3^. In that study, succinate potentiated several aspects of brain biochemistry: it significantly lowered extracellular L/P ratio, glutamate and glucose, trended towards elevating pyruvate, but did not significantly change the concentration of extracellular lactate^3^. Based on this, we proposed that succinate increases TCA cycle activity, thereby improving brain NADH/NAD^+^ redox state which aids glucose utilisation leading to increased pyruvate, and draws glutamate into the mitochondria to feed the TCA cycle.

The results from the present study support our previous findings and conclusions^3^ as we again found that succinate supplementation reduced extracellular L/P ratio and trended towards reducing brain extracellular glucose levels. We believe that these patients, especially those in which succinate administration was associated with greatest decrease in L/P ratio (and in general, had the highest L/P ratio prior to supplementation) are suffering from more pronounced mitochondrial dysfunction, whereas those patients that demonstrate only modest changes in L/P ratio may not be so afflicted by disrupted mitochondria, but instead by the other pathophysiological processes of TBI. More recent cell culture studies of succinate supplementation to stressed mixed glia show similar findings of increased pyruvate and lowering of tissue L/P ratio after succinate administration in a model of mitochondrial dysfunction^19^. Through increasing TCA cycle activity, glycolysis may also be upregulated which would lead to an increase in pyruvate, which commonly occurs in TBI patients^20,21^.

Another pathway by which succinate may in theory influence brain cells is via interaction with the succinate receptor (SUCNR1, also termed GPR91) which occurs in various tissues and cell types, including microglia, astrocytes and neurons^22^. SUCNR1 belongs to the G protein-coupled receptors (GPCRs) family, the largest group of human proteins involved in signal transduction across biological membranes^23^. SUCNR1 shows pleiotropic effects^22^. Originally an ‘orphan’ receptor with its actual ligand(s) unclear, the succinate-binding capability of GPR91 was subsequently discovered, although the actual range of ligands, their binding sites and their actions are still poorly understood. Due to the complexity of cell signalling, it is (as yet) difficult to categorise SUCNR1 (GPR91) roles. As one example, succinate binding to GPR91 has been reported to enhance post- hypoxia-ischaemia vascularization and reduce infarct size in a murine model of new-born hypoxia-ischaemia brain injury^24^.

ATP is the universal molecule of chemical energy in the human body but PCr is only found in tissues that require energy in bursts, such as muscle and brain. PCr is considered a temporal ‘buffer’ for ATP in the brain as it can donate its phosphate group to ADP via the action of creatine kinase. This rapidly regenerates ATP to allow continued cellular processes during periods of demand. PCr also diffuses more readily than ATP so acts as a spatial ‘buffer’ for high energy phosphates, shuttling them from where they are generated in the mitochondria to where they are used in the cell cytoplasm and cell membrane^25^. The general perception in phosphorus biochemistry is that when ATP synthesis is running normally, the PCr store is well-stocked; when ATP synthesis is struggling to meet demand the PCr store runs down. Absolute quantification of ^31^P species’ concentrations are difficult to achieve accurately with *in-vivo* ^31^P MRS, whereas PCr/ATP ratio is readily measurable, facilitating inter-comparisons within subjects and between subjects.

We found that succinate supplementation was associated with a considerable increase in PCr/ATP in some of our TBI patients, but this did not occur across the group so did not translate to a change in mean PCr/ATP overall in our modest-sized cohort. Many different pathophysiological processes contribute to secondary brain injury after TBI; including raised intracranial pressure, cerebral hypoperfusion, hypoxia, hypoglycaemia, neuroinflammation as well as mitochondrial dysfunction^26^. Conceivably, those patients whose PCr/ATP ratio was less responsive to succinate either suffered from more than one of these complex overlapping pathophysiological processes, or did not suffer from significant mitochondrial dysfunction, or suffered from mitochondrial dysfunction refractory to succinate supplementation. In those patients whose mitochondrial dysfunction is responsive to succinate, the metabolism of additional succinate to fumarate would generate FADH_2_, driving ATP generation via Complex II. Extra ATP produced (if not used) would be converted to the readily-mobilised store PCr, thereby elevating the PCr/ATP ratio (Fig. 3b). Importantly, those patients who did not respond to succinate at least had no significant deterioration in their metabolic profile.

An elevated L/P ratio has been shown to be independently associated with poor outcome after TBI^27^, but it is not clear if this is because of brain energy failure. The extracellular L/P ratio is thought to reflect the L/P ratio in the cytoplasm – itself in equilibrium with cytoplasmic NADH/NAD^+^^4^. Lactate and NADH accumulate when generated by glycolysis; and also by the TCA cycle if the NADH cannot be used by Complex I of the electron transport chain – the principle mechanism for cellular generation of ATP. The strong association between percentage change in L/P ratio and percentage change in PCr/ATP in our small patient group (Spearman’s rho = −0.86, p = 0.024) (Fig. 3a) supports the proposal that L/P ratio is linked to downstream energy metabolism, as well as being a representative of NADH/NAD^+^ redox state. This is the first time that tissue NADH/NAD^+^ redox, as reflected by the extracellular L/P ratio, has been shown to be linked to tissue high energy phosphates (PCr and ATP) in the human brain.

The intracellular pH measured by ^31^P MRS in this study did not correlate with succinate supplementation in the group as a whole. As this pH is intracellular, it is possible that these values might not necessarily closely reflect the L/P ratio change measured extracellularly. Intracellular pH has been shown by ^31^P MRS in rat TBI models to decrease hyper-acutely following injury, followed by a period of intracellular alkalosis^28,29^. A ^31^P MRS study of recovering patients in the subacute/chronic phase of TBI found (intracellular) alkalosis of patients’ white matter^5^. The pH changes associated with succinate in Patients A and G could conceivably be indicating a return to more normal physiology, but this would need to be corroborated with further study.

We chose to administer succinate through microdialysis catheters because of our experience using this technique and because it directly delivers succinate focally to the brain – avoiding issues with the blood-brain barrier or tissue hypoperfusion seen in the aftermath of TBI. However, the diffusion of succinate from a microdialysis catheter may be insufficient to influence the whole of our chosen MRS voxel’s metabolism. Thus, the effect of succinate will be diluted by the metabolism of unsupplemented tissue within the same voxel. The low gyromagnetic ratio of ^31^P compared to ^1^H^30^ meant that we could not decrease our voxel size any further than 2.5 × 2.5 × 2.5 cm^3^ whilst maintaining an acceptable signal-to-noise ratio within a practical scan duration (35 minutes). Intravenous supplementation of succinate is an alternative delivery method, but needs to be further studied and compared with microdialysis brain delivery.

Focal administration of succinate allows direct comparison between a region of succinate supplementation and an unsupplemented region in each patient’s brain. However, a caveat to our correlation of L/P ratio and PCr/ATP is that changes in L/P ratio are related to baseline (unsupplemented) perfusion results from the same region of brain, whereas differences in PCr/γATP are relative to an equivalent, paired frontal voxel that did not receive supplementation, scanned simultaneously. Due to clinical considerations, an acute-phase ventilated, sedated TBI patient is not scanned on consecutive days. It should be noted that the microdialysis pumps were disconnected immediately before entering the MR scanner as the pump batteries are non-MR-compliant. As microdialysis delivery of substrates occurs relatively slowly, we consider it unlikely the ca. 15-minute interval between pump disconnection and ^31^P MRS acquisition diminished the effect of perfused succinate.

Our pilot study was designed to be able to detect large changes in PCr/γATP after microdialysis delivery of succinate to the injured brain. The apparent 10.8% overall rise in PCr/γATP in our cohort did not reach statistical significance but might achieve this in future with a larger number of patients. Nevertheless, we have demonstrated a significant correlation between changes observed on focal succinate perfusion, namely percentage decrease in extracellular L/P ratio and percentage increase in brain (intracellular) PCr/ATP.

Sedation is an important part of patient management in neurocritical care during the acute phase of major traumatic brain injury. Sedation’s effects include reduction in cerebral metabolic demand and reduction in intracranial pressure^31^. Whereas we did not measure individual patients’ depth of sedation, all were sedated sufficiently for endotracheal intubation, mechanical ventilation and control of intracranial hypertension. No changes were made to their targeted depth of sedation during the study period. As each patient acted as their own control the effects of sedation would be equivalent across all ^31^P MRS voxels in that individual, and during their baseline and supplementation periods of microdialysis perfusion. Variability in time interval between patient injury and scan was due to some patients being initially less stable - so that they could not tolerate lying flat any sooner, despite optimal medical management. Thus, as all patients still required full sedation, we consider them all to be in the ‘acute phase’ of TBI, and any difference in brain metabolism due to interval from injury accounted for by each patient acting as their own internal control.

Due to the heterogeneity of TBI, it is difficult from the outset to anticipate which patients will suffer from mitochondrial dysfunction – the group we postulate will benefit most greatly from succinate supplementation. Such individuals can be identified by a combination of multimodality monitoring and advanced scanning technologies. We propose that a larger study is warranted, including intravenous infusion of succinate to address any concerns of limited diffusion from microdialysis catheters, and performing ^31^P MRS measurement before and after succinate perfusion. For reasons mentioned above, an essential prerequisite for employment of succinate is the existence of adequate oxygenation and the absence of hypoxia or ischaemia.

In conclusion, we are the first to demonstrate the effect of succinate delivery via microdialysis on the traumatised human brain’s NADH/NAD^+^ redox state (microdialysate L/P ratio, using an ISCUS bedside analyser) and brain high energy phosphate metabolism (PCr/ATP ratio, using ^31^P MRS of frontal voxels). We found that when succinate improved (lowered) brain microdialysate L/P ratio, it was also associated with an increase in brain high energy phosphate ratio. This supports the interpretation that L/P ratio is linked to brain energy state, and suggests that succinate has potential to support brain energy metabolism in patients who are suffering from mitochondrial dysfunction. Further studies of the effects of succinate on brain energy status measured by ^31^P MRS are required using larger patient groups.

## Methods

### Patient recruitment

We prospectively recruited 8 adults (aged ≥16 years) who had sustained TBI and needed intracranial monitoring, sedation, muscular paralysis, intubation and mechanical ventilation. Patients were treated in Neuro-intensive care using our standard TBI management protocols^32^. Targeted depth of sedation was not changed over the study period. Sedation was achieved with propofol, with or without midazolam. Barbiturates were not used. Electroencephalography and bispectral index were not measured as they are not routinely used in our unit. Written, informed assent was provided by patients’ relatives, and the study was carried out in conformation with the spirit and the letter of the Declaration of Helsinki. The protocol was approved by the National Research Ethics Service (NRES) Committee East of England – Cambridge Central (REC Reference No. 11/EE/0463).

### Microdialysis

Microdialysis monitoring and succinate delivery were carried out as described previously^3^. M Dialysis 71 microdialysis catheters (membrane length 10 mm, nominal molecular weight cut-off 100 kDa) (M Dialysis AB, Stockholm, Sweden) were placed via a triple lumen cranial access device (Technicam, Newton Abbot, UK) or directly at time of craniotomy/craniectomy into frontal lobe (Table 1). The catheters were not directed into, nor adjacent to, lesions identified on computerized tomography (CT), e.g. contusions. Codman Microsensor intracranial pressure (ICP) probes (Codman Neuro, Johnson & Johnson, Wokingham, UK) – and where available Licox brain tissue oxygen concentration (PbtO_2_) monitoring probes (Integra LifeSciences Corporation, Plainsboro, NJ, USA) – were inserted alongside the microdialysis catheters via the same cranial access device. ICP, CPP and PbtO_2_ was recorded and reviewed with ICM+ software (Cambridge Enterprise Ltd., Cambridge, UK)^33^. Catheters were perfused at 0.3 µL/min with CNS Perfusion Fluid (M Dialysis AB), composed of NaCl (147 mmol/L), KCl (2.7 mmol/L), CaCl_2_ (1.2 mmol/L) and MgCl_2_ (0.85 mmol/L) in water, supplemented with 12 mmol/L disodium 2,3-^13^C_2_ succinate (8 patients; isotopic enrichment 99%, chemical purity 99%) (Cambridge Isotope Laboratories, Inc, Tewksbury, MA, USA) formulated in CNS perfusion fluid (Manufacturing Unit, Department of Pharmacy, Ipswich Hospital NHS Trust, Ipswich, UK) for 24 hours before the MRS scan. Supplemented perfusion fluid was tested for purity, sterility, freedom from endotoxins and absence of pyrogenicity before release for use in patients. Before, during and after succinate perfusion, microdialysis collection vials were analysed hourly on a bedside ISCUS analyser (M Dialysis AB); employing enzymatic colorimetric assays for glucose, lactate and pyruvate. The microdialysis pumps were disconnected immediately before the patients went into the MRI scanner, although the catheters were kept *in-situ*. PbtO_2_ monitoring probes were disconnected and ICP probes coiled outside the RF head coil, as described previously^34^.

### Magnetic resonance spectroscopy

Frontal voxels supplemented with succinate were compared with a contralateral partner voxel in each patient. The contralateral frontal voxel was chosen as we deemed it the most relevant comparative volume of brain that would accommodate our 2.5 × 2.5 × 2.5 cm^3^ voxels. Voxels were inspected at time of grid positioning to ensure that they were equivalent on structural MRI sequences and free from significant injury. The microdialysis catheter tip (which contains gold) is seen on CT, and its position is also apparent on MRI. Therefore, voxel grids were positioned around patients’ catheters using their most recent CT scans co-registered to their MRI, then checked using susceptibility weighted MR sequences (SWI). We used 3T Siemens (Trio and Verio) scanners with a custom ^31^P head-coil (PulseTeq Ltd., Chobham, UK) which has a birdcage design that opens to facilitate use with TBI patients requiring ventilation; measuring ATP, PCr, Pi and other phosphorus-containing species. Spectra (Fig. 1a,b) were acquired using oblique 2-dimensional single slice chemical shift imaging (2D CSI) with 2.5 × 2.5 × 2.5 cm^3^ voxels, flip angle 90 degrees, TR 4000 ms, TE 2.3 ms, and 3000 Hz band-width (exemplified by Fig. 1c – frontal succinate and comparator contralateral voxel outlined in yellow). Repeats were performed for 30 (19 min) or 60 (35 min) averages using weighted sampling of k-space. ^31^P spectra were filtered with a 200 ms Hanning filter, fitted and peak areas computed using Siemens Syngo software. We used the γATP, as the αATP signal is incompletely resolved from nicotine-adenine dinucleotides and the βATP signal (at 3 Tesla) is on the edge of the excitation pulse bandwidth transmitted by the coil. We did not attempt to adjust ATP values for the presence of ADP as the latter is naturally much less abundant and much of it MR-invisible^25,35,36^. Brain intracellular pH was measured by ^31^P MRS by chemical shift difference between PCr and Pi using an established equation^37^. Patients also received standard ^1^H clinical MR imaging.

### Statistical analysis

In this pilot study we investigated whether substantial differences in high energy phosphate metabolism resulted from delivery of succinate to the brain via microdialysis catheters, based on previous microdialysis studies from our group (12% decrease in L/P ratio^3^). The difference in PCr/γATP between ^31^P MRS voxels perfused with succinate and their paired contralateral voxels was analysed using Wilcoxon signed rank test.

Microdialysis samples were defined as either being from a period of succinate supplementation, or from a preceding/succeeding period of baseline perfusion. The first two hours after starting or finishing succinate supplementation were considered a ‘run-in period’ or ‘washout period’ and results excluded, as in our previous study of succinate microdialysis^3^. We compared the period of succinate supplementation (mean 20 hours usable ISCUS data and after run-in/wash out exclusion) with baseline perfusion (mean 15 hours usable ISCUS data and after run-in/washout exclusion) using a linear mixed effect model (‘lmer’ package in R) which considers hourly data points rather than period mean/medians, whilst adjusting for ‘clustering’ of data, with a random y-intercept (starting point) for each subject.

Correlation between percent difference in measured L/P ratio, and difference in inter-voxel PCr/γATP was assessed using Spearman’s correlation (‘cor.test’ function in R).

All statistical analysis was performed in R (www.r-project.org) and significance (alpha) for all tests set at 0.05.

### Data availability

The datasets generated during and/or analysed during the current study are not publicly available for reasons of patient confidentiality, but anonymised data will be available from Prof Peter Hutchinson, who is the Principal Investigator of this study, on reasonable request.

## Electronic supplementary material

Supplementary Table S1 and S2
