# Supplementary material for: The effect of succinate on brain NADH/NAD+ redox state and high energy phosphate metabolism in acute traumatic brain injury
Source: Sci Rep. 2018 Jul 24;8:11140. doi: 10.1038/s41598-018-29255-3 (PMC6057963; doi:10.1038/s41598-018-29255-3)
Supplement: Supplementary file 1 — Supplementary Table S1 and S2 [file 41598_2018_29255_MOESM1_ESM.docx]

The effect of succinate on brain NADH/NAD^+^ redox state and high energy phosphate metabolism in acute traumatic brain injury.

## Authors

Matthew G. Stovell^1^*, Marius O. Mada^2^, Adel Helmy^1^, T Adrian Carpenter^2^, Eric P. Thelin^1,3^, Jiun-Lin Yan^1,4^, Mathew R. Guilfoyle^1^, Ibrahim Jalloh^1^, Duncan J Howe^5^, Peter Grice^5^, Andrew Mason^5^, Susan Giorgi-Coll^1^, Clare N Gallagher^1,6^, Michael P Murphy^7^, David K Menon^2,8^, Peter J Hutchinson^1,2^ † and Keri LH Carpenter^1,2^†

^1^Division of Neurosurgery, Department of Clinical Neurosciences, University of Cambridge, UK

^2^Wolfson Brain Imaging Centre, Department of Clinical Neurosciences, University of Cambridge, UK

^3^Department of Clinical Neuroscience, Karolinska Institutet, Stockholm, Sweden

^4^Department of Neurosurgery, Keelung Chang Gung Memorial Hospital, Chang Gung University College of Medicine, Taoyuan, Taiwan.

^5^Department of Chemistry, University of Cambridge, UK

^6^Division of Neurosurgery, Department of Clinical Neurosciences, University of Calgary, Canada

^7^MRC Mitochondrial Biology Unit, University of Cambridge, UK

^8^Division of Anaesthesia, Department of Medicine, University of Cambridge, UK

†Joint senior authors: Peter J Hutchinson and Keri LH Carpenter

## *Corresponding author:

Matthew G. Stovell, BSc, MBBS, MRCS

University of Cambridge,

Department of Clinical Neurosciences,

Division of Neurosurgery,

Box 167, Cambridge Biomedical Campus,

Cambridge,

CB2 0QQ,

UK.

Email: [mgs48@cam.ac.uk](mailto:mgs48@cam.ac.uk) Tel: +44 1223 336952 Fax: +44 1223 216926

## Supplementary Table S1. Intracellular pH results from ^31^P MRS analysis of frontal voxels supplemented with succinate, compared simultaneously to their contralateral unsupplemented voxels

| **Subject I.D.** | **pH**  **Succinate-supplemented voxel** | **pH**  **Unsupplemented (contralateral)**  **voxel** |
| --- | --- | --- |
| A | 6.920 | 7.170 |
| B | 7.060 | 7.028 |
| C | 7.118 | 7.028 |
| D | 7.012 | 6.981 |
| E | 7.110 | 7.052 |
| F | 7.170 | 7.179 |
| G | 6.951 | 7.093 |
|  |  | |
| Wilcoxon signed rank test | *p = 1* | |

^31^P MRS measurements of pH in the frontal voxels of the seven patients who received succinate supplementation. Each voxel that received succinate was matched to a partner contralateral frontal voxel that did not receive supplementation, within the same patient. The difference in pH between supplemented and matched non-supplemented voxels was not statistically significantly different (two-tailed Wilcoxon signed rank test, p = 1)

## Supplementary Table S2. Patient mean multimodality monitoring results from baseline and supplementation period of microdialysis perfusion

| **Modality** | **Condition** | **A** | **B** | **C** | **D** | **E** | **F** | **G** | **H** |
| --- | --- | --- | --- | --- | --- | --- | --- | --- | --- |
| ICP  (mmHg) | Baseline period | 9 | 12 | 14 | 10 | 14 | 18 | 19 | 12 |
|  | Succinate supp. | 9 | 13 | 15 | 10 | 16 | 19 | 15 | 14 |
| CPP  (mmHg) | Baseline period | 81 | 76 | 75 | 77 | 74 | 75 | 74 | 75 |
|  | Succinate supp. | 83 | 71 | 73 | 78 | 82 | 75 | 74 | 80 |
| PbtO_2_  (mmHg) | Baseline period | 29 | NA | NA | 31 | NA | 37 | 25 | 34 |
|  | Succinate supp. | 28 | NA | NA | 27 | NA | 56 | NA | 31 |

Mean results of intracranial pressure (ICP), cerebral perfusion pressure (CPP) and brain tissue oxygen tension (PbtO_2_) during baseline perfusion with normal CNS perfusion fluid and 12 mmol/L 2,3-^13^C_2_ succinate perfusion (Succinate supp.), recorded using ICM+.
